# Mitophagy in TGEV infection counteracts oxidative stress and apoptosis

**DOI:** 10.18632/oncotarget.8345

**Published:** 2016-03-24

**Authors:** Liqi Zhu, Chunxiao Mou, Xing Yang, Jian Lin, Qian Yang

**Affiliations:** ^1^ Key Laboratory of Animal Physiology and Biochemistry, Ministry of Agriculture, Nanjing Agricultural University, Nanjing, Jiangsu, People's Republic of China; ^2^ College of Life Sciences, Nanjing Agricultural University Weigang No.1, Nanjing, Jiangsu, China

**Keywords:** transmissible gastroenteritis virus, intestinal epithelia cells, mitophagy, ROS, apoptosis

## Abstract

The intestinal epithelial cells contain a large number of mitochondria for persisting absorption and barrier function. Selective autophagy of mitochondria (mitophagy) plays an important role in the quality control of mitochondria and maintenance of cell homeostasis. Transmissible gastroenteritis virus (TGEV) is a porcine enteropathogenic coronavirus which induces malabsorption and lethal watery diarrhea in suckling piglets. The role of mitophagy in the pathological changes caused by TGEV infection is unclear. Here, we report that TGEV induces mitophagy to suppress oxidative stress and apoptosis induced by viral infection in porcine epithelial cells (IPEC-J2). We observe that TGEV infection induce mitochondrial injury, abnormal morphology, complete mitophagy, and without obvious apoptosis after TGEV infection. Meanwhile, TGEV also induces DJ-1 and some antioxidant genes upregulation to suppress oxidative stress induced by viral infection. Furthermore, silencing DJ-1 inhibit mitophagy and increase apoptosis after TGEV infection. In addition, we demonstrate for the first time that viral nucleocapsid protein (N) is located in mitochondria and mitophagosome during virus infection or be expressed alone. Those results provide a novel perspective for further improvement of prevention and treatment in TGEV infection. These results suggest that TGEV infection induce mitophagy to promote cell survival and possibly viral infection.

## INTRODUCTION

Coronaviruses (CoVs), a genus in the coronaviridae family, are pleomorphic, enveloped viruses [1], and include alpha-, beta-, and gammacoronavirus, as well as a tentative new genus, deltacoronavirus [2]. Transmissible gastroenteritis virus (TGEV), a coronavirus in the alphacoronavirus genus, causes clinical watery diarrhea, dehydration and vomiting in piglets less than 2 weeks old [3], and persists in lung or intestine up to 104 days [4], but does not cause obvious apoptosis in intestinal epithelial cells [5]. Although intestinal epithelial cells are the targets for TGEV, little is known about TGEV infection or the mechanisms underlying intestinal injury, diarrhea, dehydration and long periods carry of virus.

Intestinal epithelial cells play an important role in the nutrition absorption and immune response against pathogens. The absorption and barrier functions are heavily dependent on mitochondrial function [6–8]. Because mitochondria also play important roles as energy sources, platforms for inflammatory and immune response signaling, and regulators of apoptosis, the mitochondrial mass, function and integrity are strictly regulated in order to respond to varying energy requirements and environmental conditions [9]. Dysfunctional or damaged mitochondria trigger a destructive cycle of mitochondrial damage and reactive oxygen species (ROS) generation, which is detrimental to cell survival [10]. Therefore, cells maintain a balance between mitochondrial biogenesis [11] and the removal of damaged mitochondria [12]. Evidence suggests that mitophagy may counteract apoptosis [13–15]. Several viruses such as Hepatitis B Virus (HBV) [16] and Hepatitis C Virus (HCV) [17] stimulate mitophagy and attenuate apoptosis, presumably to enhance viral replication. Although TGEV induces apoptosis in some cell lines, but not in intestinal epithelial cells [5], the potential role played by mitophagy in this system has not yet been explored. Here, we present experiments that examine the effect of TGEV infection on mitochondrial function and mitophagy in the nontransformed porcine intestinal epithelial cell line IPEC-J2. IPEC-J2 cell is originally isolated from jejunal epithelia of a neonatal unsuckled piglet, models *in vivo* function of the small intestine more closely than colon tumorigenic cell lines [18–21]. Our data reveal that TGEV infection promotes selective autophagic degradation of damaged mitochondria *via* mitophagy, which attenuates apoptosis and enhances viral infection.

## RESULTS

### TGEV infection induces mitochondrial damage, reduction and the formation of mitophagosome-like vesicles

**Figure 1:**
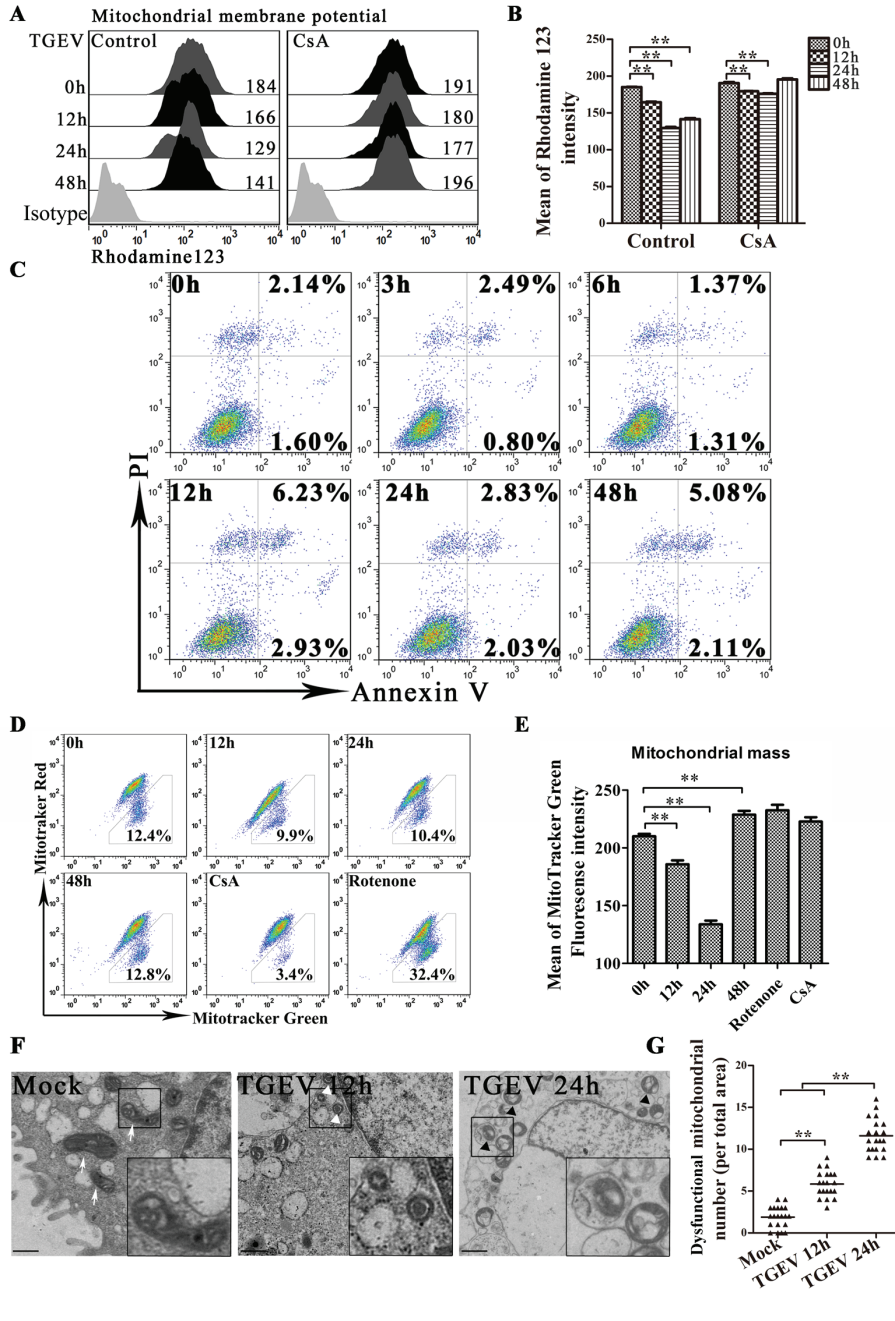
TGEV infection induces mitochondrial damage, reduction and the formation of mitophagosome-like vesicles **A.** and **B.** IPEC-J2 cells were treated with or without CsA, followed by TGEV infection for 0 h, 12 h, 24 h, and 48 h. After infection, cells were harvested, stained with Rhodamine 123, and subjected to FACS to examine mitochondrial membrane potential. Histograms representing mitochondrial membrane potential fluorescence intensity are shown in panel A and mean intensities are shown in panel B. **C.** IPEC-J2 cells were infected with TGEV for 0 h, 3 h, 6 h, 12 h, 24 h, 36 h, and 48 h, after which, Annexin V-PI double staining was performed to differentiate cells in early apoptosis (Annexin V+, PI-) from those in late apoptosis (Annexin V+, PI+). **D.** IPEC-J2 cells were infected with TGEV for 0 h, 12 h, 24 h, and 48 h. Cells treated for 12 h with CsA or Rotenone were used as negative and positive controls, respectively. Abnormal and normal mitochondria were quantitated by FACS using the fluorescent probes Mitotracker Green (stains all mitochondria) and Mitotracker Red (stains functional mitochondria). Populations are shown as dot plots. The fraction of dysfunctional mitochondria was calculated for three independent experiments as 100% × [(green stained mitochondria) - (red stained mitochondria)] / (green stained mitochondria). **E.** the statistical results of mitotracker Green in Figure 1D. **F.** 12 h and 24 h after TGEV infection, transmission electron microscopy was used to assess mitochondrial morphology. White arrows indicate normal mitochondria with clear cristae, and white triangles indicate abnormal mitochondria without clear cristae. Abnormal mitochondria in autolysosome-like vesicles were observed 24 h after TGEV infection (right panel, black triangles). Images in the black box were enlarged 2.5 times, and placed in the lower right corner of the each picture. Scale bars = 5 μm. **G.** Abnormal mitochondria (including the mitochondria in autolysosome-like vesicles) were counted in 20 cells at each time point. Data shown are the means ±SD of three independent experiments. *, *P* < 0.05; **, *P* < 0.01. The error bars represent standard deviations.

Previous study suggested that the infection of TGEV induces huge damage of mitochondrial in ST cells [22], we investigated TGEV-infected IPEC-J2 to learn if they respond similarly. As shown as in Figure 1A and 1B, the level of membrane electric potential (Δψ), decrease at 12 hours after TGEV infection, reaches a minimum at 24 hours, despite treated with ciclosporin A (CsA) or not. The decrease of Δψ could be partial suppressed by CsA (a strong stabilizer of Δψ) treatment. The reduction of membrane potential is often associated with cell apoptosis. However, the expected apoptosis does not occur after TGEV infection (Figure 1C). The total mitochondrial mass is another important factor to contribute Δψ. Using MitoTracker Green FM (total mitochondria) and MitoTracker Red CMXRos (functional mitochondria), we found that the tendency of total mitochondrial mass is similar with that of Δψ after TGEV infection (Figure 1E) and the ratio of dysfunctional mitochondria does not change significantly (Figure 1D). The decrease of total mitochondrial mass indicated mitochondria degradation, which usually performed by autophagy.

To confirm whether mitochondria were damaged and degraded by autophagy after TGEV infection, the ultrastructure of mock- or TGEV-infected IPEC-J2 cells was observed by transmission electron microscope (TEM). As shown as in Figure 1F, swollen mitochondria and mitochondria lacking cristae were observed after TGEV infection, indicative of injured mitochondria. We also observed double membrane vesicles surrounding mitochondria in TGEV-infected IPEC-J2 cells (Figure 1F). We suggest that these are autophagosome-like or mitophagosome-like vesicles. The mitophagosome-like vesicles were rarely observed in mock-infected cells (Figure 1G). The microscopy data are summarized quantitatively in Figure 1F, which shows the numbers of dysfunctional mitochondria observed in mock and infected cells.

Together, these data suggest that TGEV infection induce mitochondrial damage and may induce selected elimination of damaged mitochondria by autophagy.

### TGEV infection induces the accumulation of autophagosomes and preserves autophagic flux

**Figure 2:**
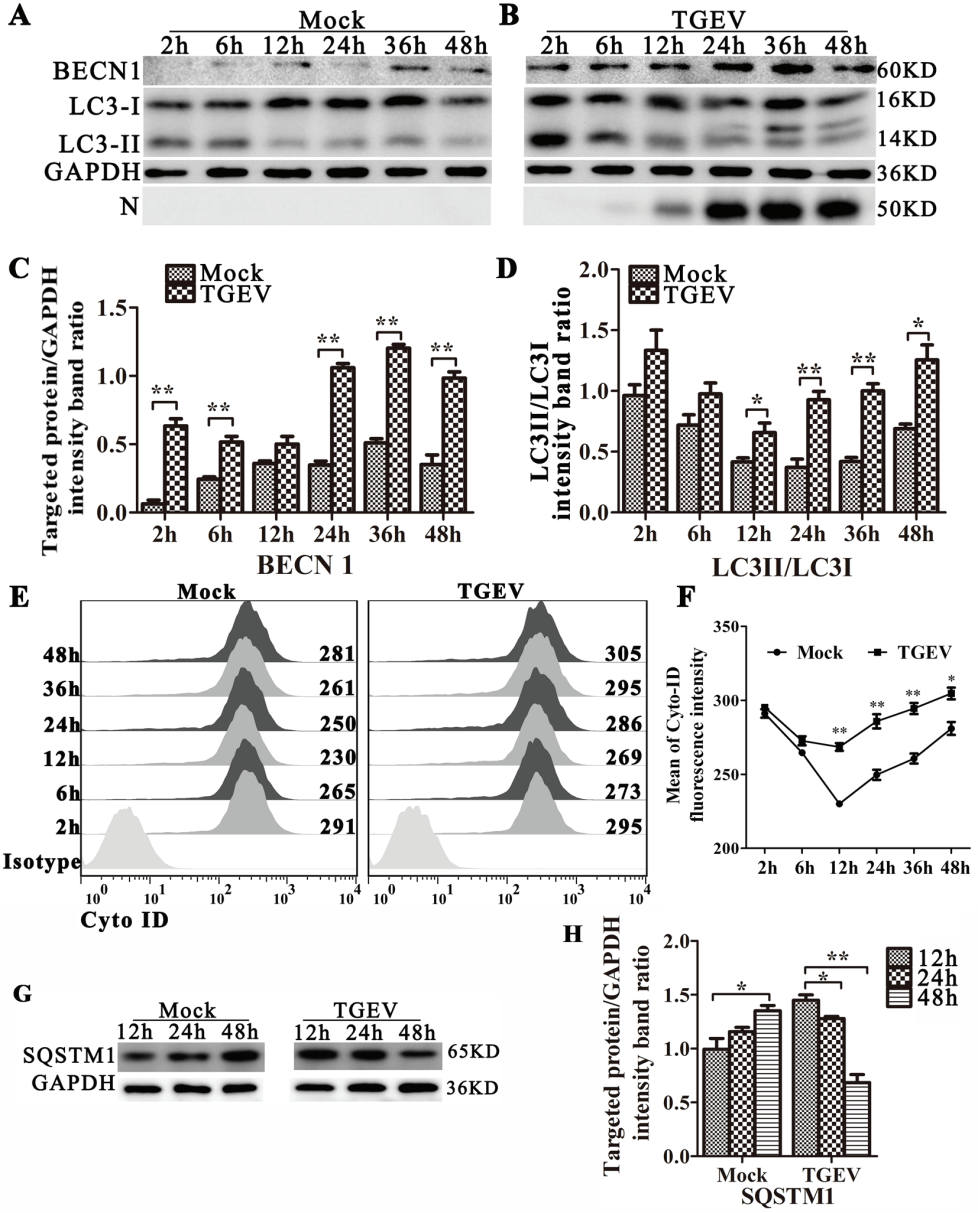
TGEV infection induces autophagy and promotes autophagic flux IPEC-J2 cells were mock-infected **A.** or TGEV-infected **B.** for 2 h, 6 h, 12 h, 24 h, 36 h, and 48 h. The expression of BECN1, LC3-I, LC3-II, GAPDH (loading control), and N were analyzed by western blot using the specific antibodies as described in Materials and Methods. Relative BECN1 and LC3-II/LC3-I levels are presented in **C.** and **D.**, respectively. E and F: IPEC-J2 cells were mock-infected (left panel) or TGEV-infected (right panel) for 2 h, 6 h, 12 h, 24 h, 36 h, and 48 h, then stained using Cyto-ID Green Detection Reagent and subjected to FACS analysis. Panel **E.** shows the distribution of fluorescence intensity, and panel **F.** shows the fluorescence at each time point, calculated as mean ± SD for three independent experiments. One-way ANOVA; *, *P* < 0.05; **, *P* < 0.01. **G.** IPEC-J2 cells were mock-infected or TGEV-infected for 12 h, 24 h, and 48 h. Cell proteins SQSTM1 and GAPDH were then analyzed by western blot with anti-SQSTM1 and anti-GAPDH (loading control) antibodies. **H.** Statistical results of SQSTM1. The data represent the mean ± SD of three independent experiments. One-way ANOVA; *, *P* < 0.05; **, *P* < 0.01.

To determine if autophagy is triggered by TGEV infection, we examined the levels of the autophagy marker (LC3 conversion) in TGEV-infected cells (Figure 2A and 2B). We also examined the levels of beclin 1(BECN1) expression, an important regulator in autophagy, in TGEV-infected cells (Figure 2A and 2B). TGEV nucleocapsid protein (N) was used to monitor the progression of infection. LC3 (microtubule associated protein 1 light chain 3) is a marker for assessing autophagy and correlates well with the formation of the autophagosome [23]. Our results show that LC3-II/LC3-I is more abundant in TGEV-infected IPEC-J2 cells relative to levels in mock-infected cells. BECN1, the initial step of the autophagy pathway [24], is also over-expressed in TGEV infected cells (Figure 2C and 2D). Meanwhile, we detected autophagy by Cyto-ID Green Detection Reagent, which is induced at 12 h to 48 h post-infection (Figure 2E and 2F).

To determine whether a complete autophagic response is triggered by TGEV infection, we used western blot analysis to measure the degradation of SQSTM1 (p62), a marker for autophagy-mediated protein degradation pathway. As shown in Figure 2G and 2H, TGEV-infected cells have lower levels of SQSTM1 protein at 12 h to 48 h post-infection, compared with mock-infected cells. We also examined autophagic flux by using fluorescence microscopy with pLVX-mRFP-EGFP-LC3 lentiviral transfected cells (Figure S1). In TGEV-infected cells, mRFP puncta have accumulated in the cytoplasm at 12 h to 48 h post-infection, whereas EGFP puncta are weak. A similar response is seen in cells treated with rapamycin, indicating that complete autophagic flux is induced by TGEV infection. It is worth noting that chloroquine (CQ), an inhibiter of lysosomal degradation, could induce incomplete autophagy with or without Rapamycin in IPEC-J2 cells. Together, these data show that a complete autophagic response is induced in IPEC-J2 cells following TGEV infection.

### TGEV infection induces complete mitophagy

**Figure 3:**
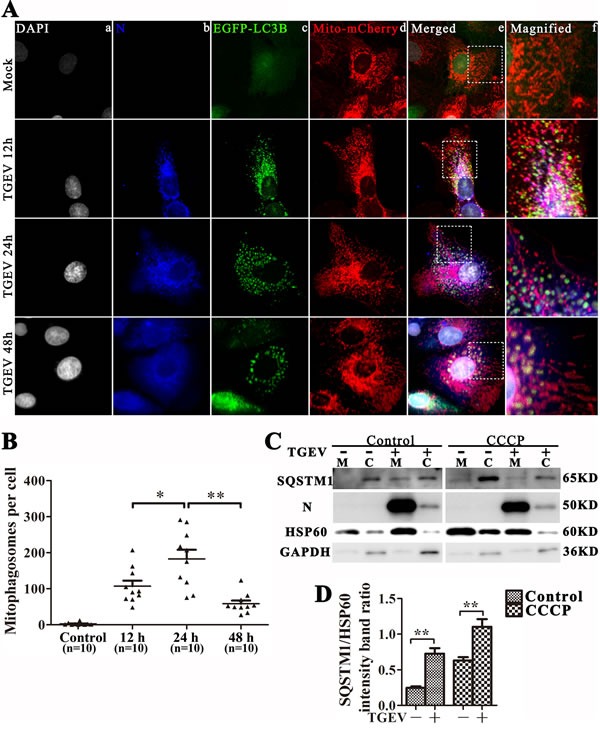
TGEV infection induces mitophagy **A.** IPEC-J2 cells were infected with TGEV for 0 h, 12 h, 24 h, and 48 h, then fixed and analyzed for indirect immunofluorescence using an anti-TGEV N protein antibody, followed by the corresponding secondary antibodies conjugated to Alexa Flour 647, as described in Materials and Methods. Cell nuclei were counterstained with DAPI and fluorescence signals were visualized by confocal immunofluorescence microscopy. In the images, the colors correspond to nuclei (white), N (blue), EGFP-LC3 (green), and mito-mCherry (red). Co-localized signals for N, EGFP-LC3 and mitochondria are white in the cytoplasm of the merged images. Higher magnification images represent the regions enclosed in white squares. The co-location of EGFP and mCherry punctra was counted and showed in **B. C.** IPEC-J2 cells were pre-treated with or without CCCP, followed by mock or TGEV infection for 24 h. SQSTM1, N and HSP60 were then detected by western blot in mitochondrial or cytoplasmic cell fractions. mtHSP60 and GAPDH were used as loading controls for mitochondrial and cytoplasmic fractions, respectively. **D.** Quantitative results for SQSTM1 in mitochondria fraction, the data represent the mean ± SD of three independent experiments. One-way ANOVA; *, *P* < 0.05; **, *P* < 0.01.

The degradation of damaged mitochondrial in autophagosomes is critical for mitophagy and mitochondrial turnover [25]. To examine whether TGEV infection could induce mitophagy, we constructed the multi-function lentiviral vector pLVX-EGFP-LC3B-IRES-mito-mCherry and transfected it into IPEC-J2 cells. After TGEV infection, EGFP-LC3B clearly translocates to mitochondria 12 h and 24 h after infection, but co-localization of EGFP-LC3B and mito-mCherry decreases after 48 h (Figure 3A and 3B). To examine whether mitophagic flux is complete, a sensitive dual fluorescence reporter expressing mRFP-EGFP-mito fused in-frame to a mitochondrial targeting sequence (transmembrane structure of Bcl-xL) was employed to observe the completion of the mitophagic process by delivery of the engulfed mitochondria to lysosomes for degradation. As show as in Figure S2, lots of mitochondria present as puncta, with weak green florescent and bright red florescent, especially at 24 h and 48 h post TGEV infection. Combined with the complete autophagic flux (Figure 2G, 2H and Figure S1), we presumed the mitophagic degradation is not inhibited by TGEV infection.

Surprisingly, TGEV N protein is observed in mitochondria and mitophagosomes during infection. To confirm the location of autophagic proteins and N, mitochondrial and cytoplasmic proteins were separated and subjected to western blot analysis. Result displayed that protein N is highly enriched in the mitochondrial fraction (Figure 3C). In addition, we found that SQSTM1, a mediator of mitophagy [26] is relatively more abundant in mitochondria after TGEV infection (Figure 3C and 3D). To examine the location of N on mitochondrial dependent viral infection, we constructed the lentiviral vector pLVX-N-Flag (resistance to puromycin) and transfected to IPEC-J2 cells. The co-localization with N-Flag and mitochondria is presented in Figure S3A. Moreover, the morphology of mitochondria in N-Flag cells is different with control cells (Figure S3A). In addition, the ROS and mtROS (Figure S3B and S3C) is also increased, but does not cause obvious apoptosis (Figure S3D) by N-Flag protein expression. The total mitochondrial mass is also decreased (Figure S3E) in N-Flag cells, which indicate mitophagy may be actived. To confirm that, we constructed the pLVX-EGFP-LC3B (resistance to blasticidin) lentiviral and transfected to N-Flag cells. As show as in Figure S3G, the co-localization of mitochondria, N-Flag and EGFP-LC3B puncta is presented in N-Flag cells. Together, these data indicate that the nucleocapsid protein of TGEV may contribute to mitochondrial dysfunction and induces mitophagy in IPEC-J2 cells.

### Mitophagy enhances TGEV infection by mitigating cell apoptosis

**Figure 4:**
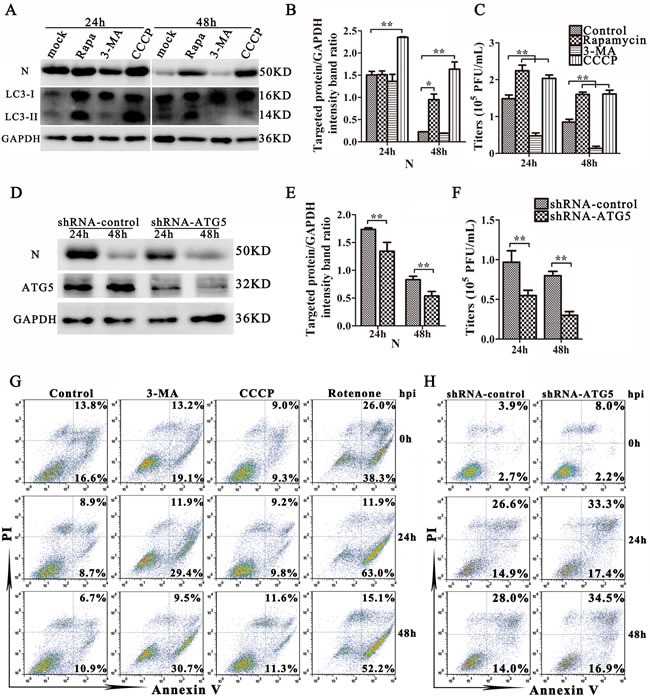
Mitophagy enhances TGEV infection **A.** IPEC-J2 cells were treated with Rapamycin, 3-MA, CCCP, or mock and then infected with TGEV for 24 h and 48 h. Viral infection results are presented in **B.** and **C. D.** IPEC-J2 cells were transfected with shRNAs targeting ATG5 or control (scrambled) shRNAs, following infection with TGEV as in (A). Viral infection results are presented in **E.** and **F.** The expression of LC3, N, and GAPDH (loading control) were analyzed by western blot with specific antibodies as described in Materials and Methods. At 24 h and 48 h post infection, the extracellular virus titers were measured by plague assay. **G.** Apoptosis levels in control, 3-MA, CCCP and Rotenone treated cells were evaluated by FACS at 0 h, 24 h and 48 h post infection with TGEV. Annexin V-PI double staining was performed to differentiate cells in early apoptosis (Annexin V+, PI-) from those in late apoptosis (Annexin V+, PI+) stages. **H.** Apoptosis levels in control or ATG5 knockdown cells were evaluated by FACS at 0 h, 24 h and 48 h post infection with TGEV. The data represent the mean ± SD of three independent experiments. One-way ANOVA; *, *P* < 0.05; **, *P* < 0.01.

To explore the role of autophagy in TGEV infection, IPEC-J2 cells were treated with rapamycin or CCCP, and then infected with TGEV. The expression of N was monitored as a proxy for viral infection. An increase in N expression was found in both rapamycin- and CCCP-treated cells at 48 h post-infection (Figure 4A and 4B). In the same experiment, cells were also exposed to 3-MA, which inhibits autophagy by blocking the formation of autophagosomes [27, 28]. 3-MA treatment reduced the expression of LC3-II and N at 48 h post-infection (Figure 4A and 4B) and decreased virus titer both at 24 h and 48 h post-infection (Figure 4C).

Our results using pharmacological regulators support that autophagy may increase TGEV infection. To examine the relationship between autophagy and virus infection further, shRNA knockdown experiments were performed to specifically deplete endogenous ATG5 protein. As shown in Figure 4D and 4E, IPEC-J2 cells transfected with shATG5 exhibit significantly decreased levels of endogenous ATG5 protein compared with cells transfected with control (scrambled) shRNA. Importantly, suppression of ATG5 expression reduced the expression of N and the viral progeny yield in TGEV infected IPEC-J2 cells compared with the control shRNA transfection (Figure 4F).

Apoptosis is suppressed by mitophagy by a wide variety of viruses during infection [16, 29–33]. To determine whether mitophagy attenuates apoptosis during TGEV infection, IPEC-J2 cells were simultaneously stained with Propidium Iodide (PI) and Annexin V at 0 h, 24 h, and 48 h post-infection, and analyzed by flow cytometry. The apoptosis of IPEC-J2 cells treated with CCCP was similar to that observed in untreated cells. In contrast, 3-MA treatment (Figure 4G) and ATG5 knockdown (Figure 4H) both increased apoptosis in infected cells. However, the moderate increase of apoptosis by inhibition of autophagy, drug or knock-down, indicate autophagy play multiple roles in promotion TGEV infection. Finally, TGEV infection increased early apoptosis and inhibited late apoptosis when IPEC-J2 cells were pre-treated with Rotenone (Figure 4G), a drug that stimulates apoptosis by increasing mitochondrial ROS. Together, these experiments demonstrate that mitophagy promote the infection of TGEV partly by attenuating cell apoptosis.

### Mitophagy attenuates cell apoptosis by eliminating ROS

**Figure 5:**
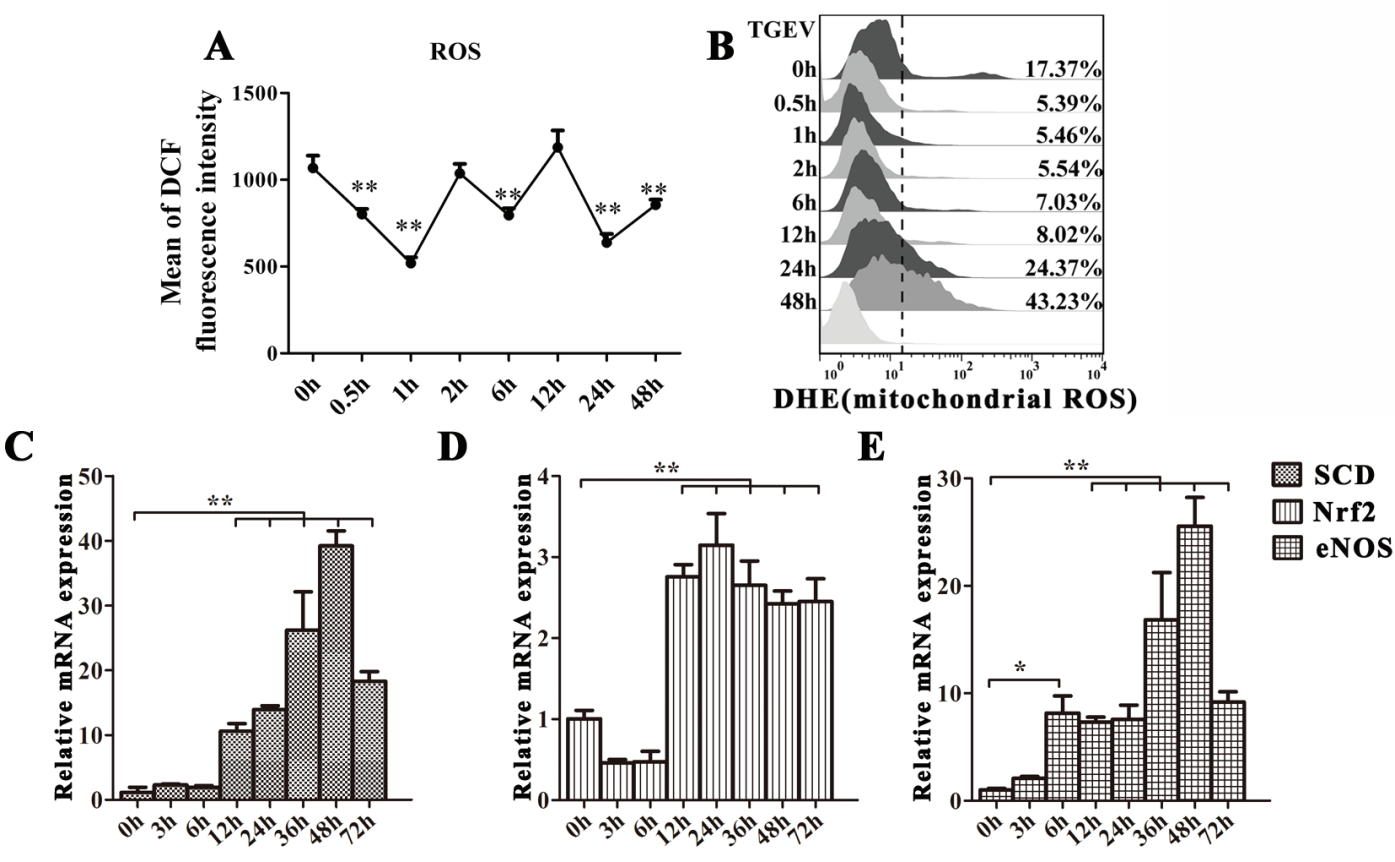
TGEV infection induces moderate oxidative stress in IPEC-J2 cells **A.** and **B.** IPEC-J2 cells were infected with TGEV for 0 h, 0.5 h, 1 h, 2 h, 6 h, 12 h, 24 h, and 48 h. The ROS and mtROS levels in cells were detected by DCFH-DA and DHE fluorescent probes and quantified with FACS. **C.**, **D.**, and **E.** IPEC-J2 cells were infected with TGEV for 0 h, 3 h, 6 h, 12 h, 24 h, 48 h, and 72 h. SCD and Nrf2 mRNA expression was significantly upregulated from 12 h to 72 h, while eNOS mRNA expression was significantly upregulated from 6 h to 72 h post infection. The data represent the mean ± SD of three independent experiments. One-way ANOVA; *, *P* < 0.05; **, *P* < 0.01.

Since the damaged of mitochondria may release ROS to induce mitophagy or apoptosis, we measured ROS levels using Dichlorofluorescein Diacetate (DCFH-DA) staining and FACS (Figure 5A). Our data show that ROS levels is fluctuated without abnormally high level. We also examined mitochondrial ROS (mtROS) production during TGEV infection by measuring the fluorescence of DHE. Between 0.5 h to 12h after infection, mtROS production decreased, but was restored and increased after 24 h (Figure 5B). To further examine oxidative stress status, we measured mRNA levels for three proteins known to be involved in modulating ROS levels: As shown as in Figure 5C–5E, the expression of SCD and eNOS begins to increase 12 h after TGEV infection, and reaches the highest level at 48 h (*P* < 0.01). The expression of Nrf2 decreases at 3 h and 6 h after TGEV infection and then increases from 12 h to 72 h, with a maximum at 24 h (*P* < 0.01).

**Figure 6:**
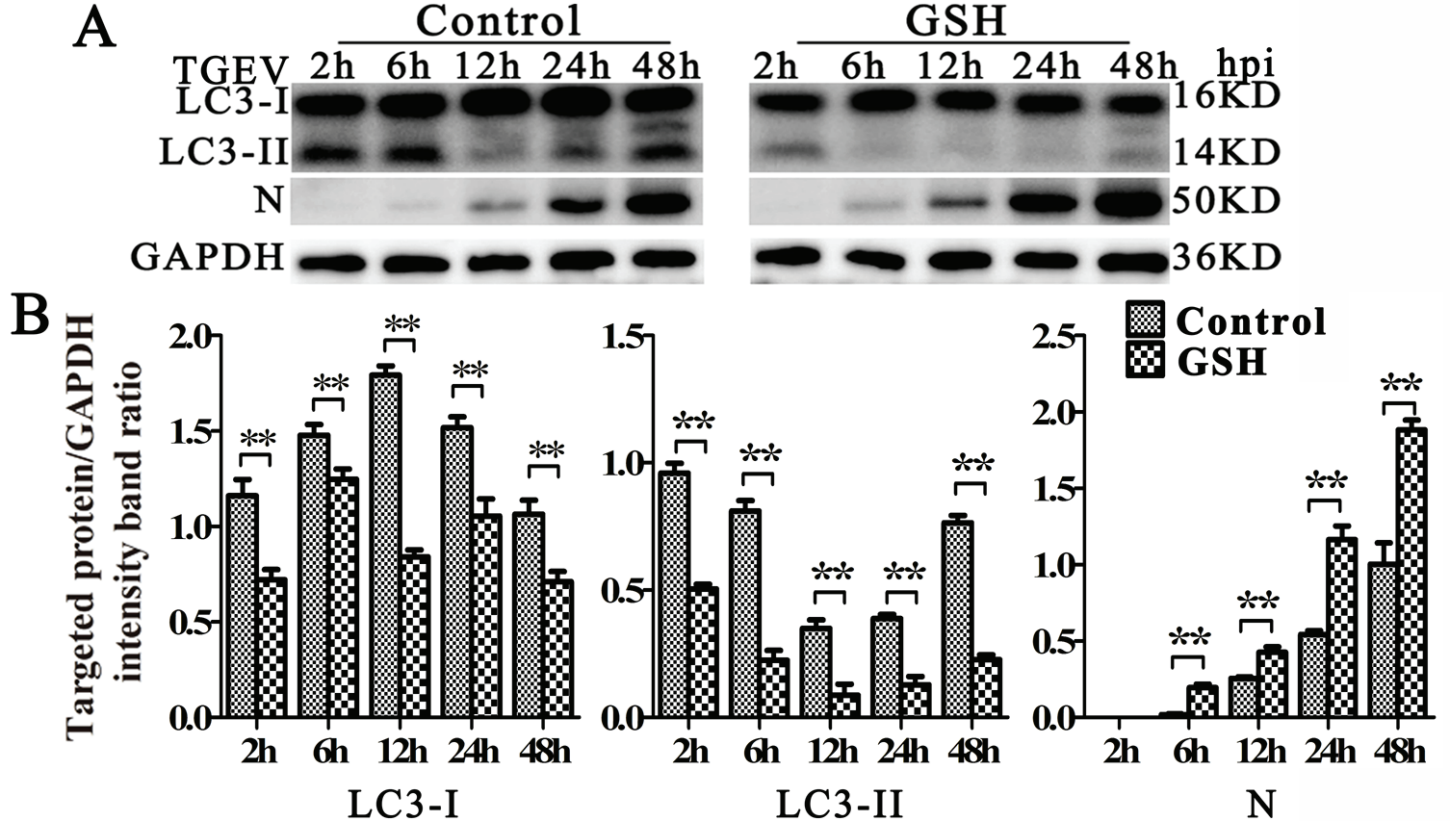
GSH suppress autophagy induced by TGEV **A.** IPEC-J2 cells were pre-treated with GSH for 12 h, then infected with TGEV for 2 h, 6 h, 12 h, 24 h, and 48 h. The expression of LC3, N, and GAPDH (loading control) were analyzed by western blot with specific antibodies as described in Materials and Methods. **B.** statistical results of LC3-I, LC3-II and N expression. The data represent the mean ± SD of three independent experiments. One-way ANOVA; *, *P* < 0.05; **, *P* < 0.01.

**Figure 7:**
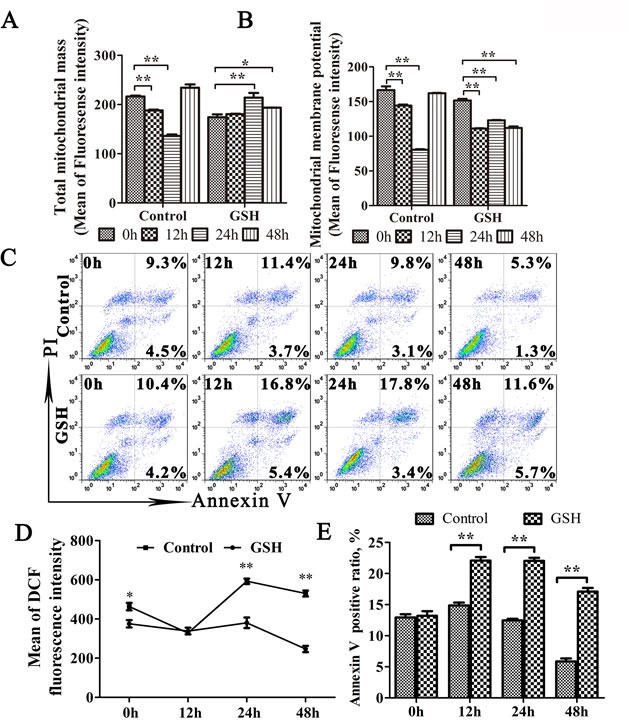
GSH suppress mitophagy and promote apoptosis **A.** to **E.** IPEC-J2 cells were GSH or mock treated for 12 h, followed by TGEV infection for 12 h, 24 h, and 48 h. Then, cells were divided into 4 equal parts, which were stained by MitoTracker Green, Rhodamine 123, DCFH-DA and Annexin V/PI, respectively. Finally, the corresponding test was carried out by flow cytometry. The mitochondrial mass, membrane potential and ROS level were presented in (A), (B) and (D), respectively. The scatter plot of apoptosis was presented in (C), the histogram of Annexin V positive cells was presented in (E). The data represent the mean ± SD of three independent experiments. One-way ANOVA; *, *P* < 0.05; **, *P* < 0.01.

Since oxidative stress plays important roles in mitophagy [34, 35], we then explored the relationship between oxidative stress and mitophagy during TGEV infection. As shown as in Figure 6A and 6B lift and middle panel, the expression of LC3-I, LC3-II decreased and N expression increased at 6 h, 12 h, and 24 h after TGEV infection in GSH-treated cells (Figure 6A and 6B right panel). To exclude the possibility of decreased mitochondrial injury by GSH, mitochondrial membrane potential and mitochondrial mass were monitored. Results (Figure 7A and 7B) show that, in GSH-treated cells, the mitochondrial membrane potential also decreases after TGEV infection, but the total mitochondrial mass increases after TGEV infection, indicating the quality of mitochondria decreased and mitophagic degradation inhibited.

Antioxidant treatment generally efficiently suppress apoptosis [36]. However, as shown in Figure 7C and 7E, the apoptosis ratio has a weak increase after TGEV infection in GSH pre-treated cells. According to the pro-apoptotic effect of ROS eliminated by GSH, the weak increase of apoptosis may be induced by other pro-apoptotic substance released by the abnormal mitochondria. These results indicated that oxidative stress is an important factor in the mitophagy induced by TGEV infection.

### DJ-1 play an important role in TGEV-induced mitophagy and viral infection

**Figure 8:**
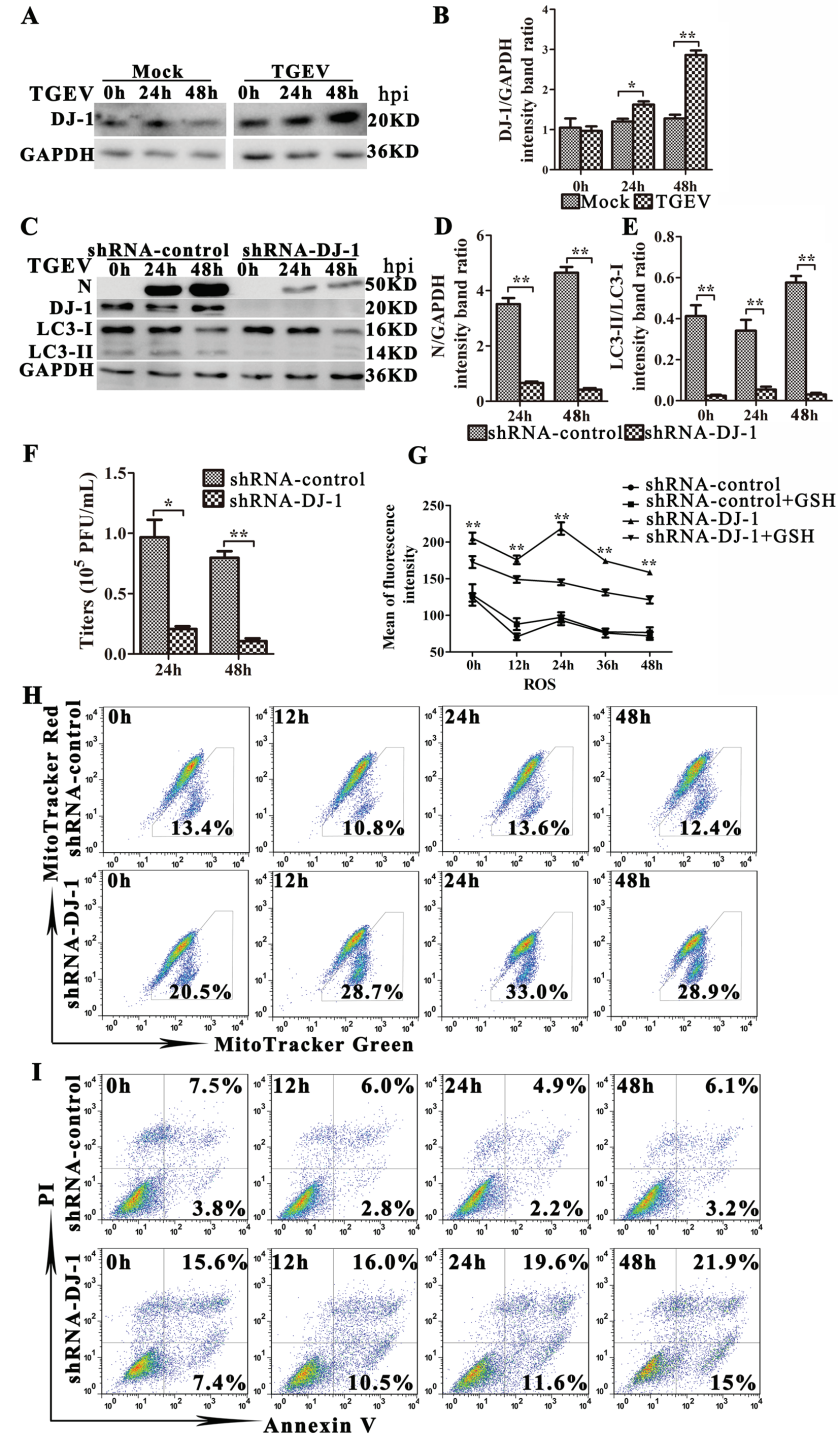
DJ-1-mediated mitophagy controls ROS release in TGEV-infected cells **A.** Cells were mock or TGEV infected for 0 h, 24 h or 48 h. The expression of DJ-1 and GAPDH (loading control) were analyzed by western blot with specific antibodies as described in Materials and Methods. **B.** Present the statistical results of DJ-1/GAPDH. **C.**-**F.** IPEC-J2 cells were transfected with shRNAs targeting DJ-1, or control (scrambled) shRNAs, and then cells were mock or TGEV infected for 0 h, 24 h or 48 h. **C.** The expression of N, DJ-1, LC3-I, LC3-II, and GAPDH (loading control) was analyzed by western blot with specific antibodies as described in Materials and Methods. **D.** and **E.** Present the statistical results of N/GAPDH and LC3-II/LC3-I. **F.** At 24 h and 48 h TGEV infection, the extracellular virus titer measured by plague assay. **G.** IPEC-J2 cells were transfected with control (scrambled) shRNAs, or shRNAs targeting DJ-1, then mock or GSH treated for 12 h, followed by infection with TGEV for 0 h, 12 h, 24 h, and 48 h. The ROS level in cells was detected by the DCFH-DA fluorescent probe and quantified with spectrometry. **H.** and **I.** IPEC-J2 cells were transfected with control (scrambled) shRNAs, or shRNAs targeting DJ-1, and then infected with TGEV for 0 h, 12 h, 24 h, and 48 h. **H.** The abnormal mitochondrial ratio in cells was detected with Mitotracker Green and Mitotracker Red. **I.** Apoptosis was detected with Annexin V-PI fluorescent probes. Quantification was done by FACS. The data represent the mean ± SD of three independent experiments. One-way ANOVA; *, *P* < 0.05; **, *P* < 0.01.

DJ-1, a multifunctional redox-sensitive protein, is associated with the oxidative stress cell death cascade [37–40]. The loss of DJ-1 contributes to mitochondrial injury and apoptosis in many mammalian cells [40–42]. We found that the expression of DJ-1 increased significantly 24 h and 48 h post TGEV infection (Figure 8A and 8B). To determine whether DJ-1 mediates mitophagy during TGEV infection, we decreased DJ-1 gene expression in IPEC-J2 cells by using a lentiviral vector, pLVX-shRNA-DJ1. As shown in Figure 8C and 8E, DJ-1 knockdown decreases the expression of LC3-II significantly. Importantly, suppression of DJ-1 expression strongly reduces the expression of viral protein N and the viral progeny yield in TGEV infected IPEC-J2 cells compared with controls (Figure 8D and 8F). In contrast, the ROS level in DJ-1 knockdown cells is extremely high during TGEV infection compared with controls (Figure 8G). Additionally, GSH treatment partially relieves oxidative stress in DJ-1 knockdown cells, but fails to do so in control cells (Figure 8G). The dysfunctional mitochondrial ratio in DJ-1 knockdown cells is nearly double than observed in control cells before TGEV infection, and triples 24 h after TGEV infection (Figure 8H). Finally, apoptosis is detected in pLVX-shRNA-DJ-1 transduction cells after TGEV infection. The apoptosis increased significantly in DJ-1 knockdown cells compared with controls at 12 h, 24 h, and 48 h after TGEV infection (Figure 8I). These results suggest that DJ-1 play an important role in suppression oxidative stress and induction mitophagy during TGEV infection.

## DISCUSSION

Our study demonstrates for the first time that TGEV provokes mitophagy in intestinal epithelial cells. Using *in vitro* experiments, we have shown that TGEV infection induces DJ-1-mediated mitophagy to counteract oxidative stress and confers protection against apoptotic cell death in virus-infected intestinal epithelia cells. This is also likely to facilitate persistent virus infection, and may be one reason why the virus is carried for long periods in recovered pigs.

Recent evidence suggests that autophagic digestion of mitochondria is a selective process [43, 44]. Despite of the powerful function in energy generation, mitochondria could release a lot of pro-apoptotic factors, such as ROS and cytochrome c, after injury [45]. Thus, the moderate and timely mitophagy promotes cell survival and homeostatic by regulating of energy generation and by clearing dysfunctional mitochondria that signal for apoptosis. In addition, mitophagy is essential for mitochondrial turnover [46] to avoid ATP exhausting, which will consequently leading to necrosis [47]. In our study, the decrease in mitochondrial mass and increase in SQSTM1 in the mitochondrial cell fraction further supports the emergence of mitophagy after TGEV infection. The number of mitochondria within cells is regulated by the balance between mitochondrial biogenesis [48] and the removal of damaged mitochondria. Damaged mitochondria, as well as other impaired organelles and proteins in cells are degraded by specific autophagy-lysosome pathway [11]. Using the dual fluorescence reporter lentivirial pLVX-mRFP-EGFP-Bcl-xL, we demonstrated that TGEV induces complete mitophagy evident by fusion of mitophagosome with lysosome. Furthermore, CCCP treatment induces mitophagy by decreasing mitochondrial membrane potential [49], causing mitochondrial fragments to appear in the cytoplasmic fraction (Figure 3C). In contrast, TGEV infection decreased mitochondrial fragments in the cytoplasm fraction in CCCP-treated cells, which suggests the promotion of autophagic degradation was triggered by TGEV. These results indicate that TGEV infection induces complete mitophagy to eliminate abnormal mitochondria in IPEC-J2 cells, enhancing mitochondrial turnover. Although, It is reported that, the immunofluorescence could be progressed after transfected with dual fluorescence reporter mito-mRFP-EGFP [16], but we found the fluorescent intensity of EGFP was recovered after immunofluorescence (data not show). Therefore, the model of mRFP-EGFP fused with Bcl-xl transmembrane structure is only suitable for living cells. In general, the inhibition of apoptosis is a common strategy to persist viral infection in many chronic virus diseases [29, 33, 50]. As mitochondria is regarded to function as the control point in apoptotic pathways [51], it is plausible that mitophagy may play a role in the modulation of apoptosis. Most notably, mitochondrial is the main ROS factory, which functions as a signaling molecule through oxidation of redox-sensitive cysteine residues and inhibition of protein phosphorylation [52]. Because ROS can function as a destructive agent at high levels, resulting in cell death [53–55], cellular ROS homeostasis is tightly regulated. A relationship between TGEV infection and ROS production has been reported [56] but infection did not induce a significant increase of ROS in IPEC-J2 cells. These results are in contrast with those reported for ST cells after TGEV infection [22]. Due to the strong energy and material basis metabolism and the large number of mitochondria [57], the intestinal epithelial cells have the great potential to produce ROS leading to the powerful ability to control oxidative stress. In our experiments, the expression of antioxidative genes indicate that oxidative stress is probably induced by TGEV infection, which may explain the moderate ROS level. In addition, autophagy is required to degrade ROS-producing organelles such as mitochondria and peroxisomes [58]. Mitochondrial ROS (mtROS) activates general mitophagy to suppress oxidative damage [59]. We find that the peak of TGEV virus infection is accompanied by ROS or mtROS accumulation and mitochondrial degradation, but apoptosis is nearly undetectable. Obvious mitophagy can be observed 12 h after TGEV infection, and can be suppressed by GSH treatment. Many reports have shown that GSH or other antioxidants treatment could effectively inhibit apoptosis caused by oxidative stress [60–62]. The impaired mitochondria could release ROS and other pro-apoptotic substances, such as cytochrome c, etc., to promote apoptosis. The inhibition of autophagy by GSH, suppress the removing of damaged mitochondria, which may release other pro-apoptotic substances, and promote the apoptosis after TGEV infection. While, the suppressing ROS by antioxidants also inhibits apoptosis. The final effect of GSH on cell apoptosis may be combined with the inhibition of mitophagy and elimination of abnormal ROS. These data suggest that oxidative stress play an important role in mitophagy and apoptosis post TGEV infection.

Autophagy is an effective way to resist viral invasion [63] and replication [64–67]. The immune response, especially Type I IFN responses, promote autophagy to inhibit virus replication [68]. However, some viruses can highjack the autophagic structure as viral replication sites [66, 67]. Accumulated studies indicate that many coronavirus utilise the autophagy membrane structures to replication [69]. Moreover, SARS-CoV ORF9b could localize to mitochondria and induce autophagy, which caused the MAVS signalosome degradation to further suppress antiviral transcriptional responses [70]. TGEV coronavirus contains 4 major structural proteins: spike (S), envelope (E), membrane (M), and nucleocapsid (N) proteins. Previous study has demonstrated that, the nucleocapsid protein of CoVs facilitated virus RNA switching and was required for efficient transcription [71]. Moreover, TGEV nucleocapsid protein could interact with EF1a and facilitate viral replication [72]. Coronavirus nucleocapsid could localize in cytoplasm and nucleus [73–75]. While, in our results, the subcellular localization of TGEV nucleocapsid was mainly found in mitochondria, which was inconsistent with different studies [76, 77]. Although, the ROS and mtROS are elevated, distinct fragmented mitochondrial morphology is observed, and the total mitochondrial mass is decreased by N protein expression, but the mitochondrial membrane potential is not changed (Figure S3). These results indicate the balance of mitochondrial quality and quantity in IPEC-J2 cells is very successful. Despite the N protein localizing on almost mitochondria (Figure S3A), the number of mitophagosames are limited (Figure S3G). These results indicate the mitochondrial localization of N protein may create same injury but be not directly related to the mitophagy. The directly inducer may be the injured mitochondria, which is eliminated by fission form normal mitochondrial and degraded by mitophagy. Together, the nucleocapsid of TGEV may contribute to the mitochondrial injury and induction of mitophagy during TGEV infection.

Mitochondrial dysfunction is monitored by several proteins, such as NIX, BNIP3, PINK1, parkin, SQSTM1 (p62) [31, 78, 79]. Recent work reveals that DJ-1 (PARK7) acts as a signaling hub through its ability to recognize oxidative damaged in mitochondria and induce mitophagy, thus eliminating damaged mitochondria in mammalian cells [39, 80–82]. The role of DJ-1 in the regulation of oxidative stress has been extensively studied in neurodegenerative diseases [82–84]. In addition, DJ-1 plays an important role in reducing inflammation and antioxidation in gastrointestinal mucosa [85]. Despite of much work for DJ-1 in regulation of oxidative stress and mitophagy, the role of DJ-1 in autophagy/mitophagy is poorly understood. Our work indicates that mitophagy benefits TGEV infection by eliminating damaged mitochondria, thereby controlling ROS release and blocking oxidative damage. TGEV infection increases DJ-1 expression, and DJ-1 knockdown reduces autophagy and mitophagy, causing an increase in apoptosis and a decrease in TGEV infection. Furthermore, mitochondria become vulnerable in IPEC-J2 cell after DJ-1 knockdown, especially after infection with TGEV, and ROS release increases significantly, which is partly counteracted by GSH treatment. Our results are consistent with recent work in DJ-1^−/−^ mice exhibit increased sensitivity to oxidative stress [86], and glutathione or NAC treatment attenuates DJ-1-deficiency-induced phenotypes [82]. Given that DJ-1-mediated mitophagy is required to prevent TGEV-induced apoptosis, and thus favors viral infection and persistence.

**Figure 9:**
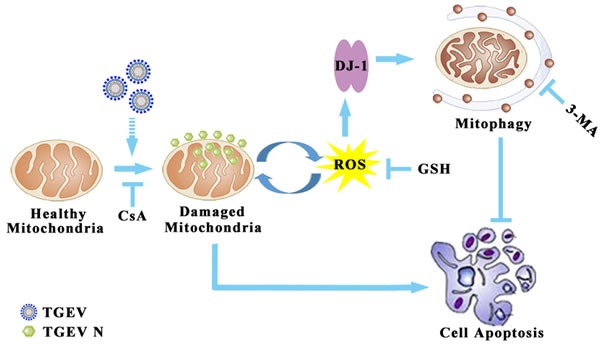
Model of mitophagy in TGEV infection The infection of TGEV first induced mitochondrial membrane potential decrease and mitochondrial injury. Then, the damaged mitochondria release ROS to trigger DJ-1 mediated selective autophagy to eliminate injured mitochondria. The ROS suppression of by GSH could inhibit mitophagy, but failed in resistance to apoptosis. In conclusion, TGEV-induced mitophagy promote cell survival and facilitate of viral infection and persistence.

In summary, this study provides evidence for the likely involvement of TGEV-induced mitophagy in facilitating the infection and the pathogenesis of gastrointestinal disease associated with infection (Figure 9) *in vitro*. Our study demonstrates for the first time that the nucleocapsid protein of TGEV is located in mitochondria. More research is needed to explain the association between mitophagy and TGEV infection, especially *in vivo*. A closer look at the relationship between the mitochondrial location of nucleocapsid protein and the elimination of dysfunction mitochondria may provide new strategies for the control of viral infection.

## MATERIALS AND METHODS

### Cells, antibodies and reagents

IPEC-J2 cell lines (Guangzhou Jennio Biotech Co, Ltd., China) used in this study were cultured in Dulbecco's Modified Eagle's Medium nutrient (DMEM from Life Technologies, Shanghai, China) supplemented with 10% fetal bovine serum (FBS, Life Technologies), 16 mM HEPES (Life Technologies), and 100 μg/mL penicillin-streptomycin (Life Technologies), and incubated in an atmosphere of 5% CO_2_ at 37°C. Cells were routinely seeded at a density of 2 × 10^5^/mL in plastic tissue culture flasks (25 cm^2^ flasks, Corning, Shanghai, China) and passaged every 3-4 days for a maximum of 20 passages. In our experiments, IPEC-J2 cells were grown on 24-,6- well or 100 mm plastic tissue culture plates (Corning) at a density of 3 × 10^5^/well, 1.5 × 10^6^/well or 7.5 × 10^6^/well, respectively.

Antibodies used in this study were: anti-LC3B (Beyotime Institute of Biotechnology, Haimen, China), anti-p62/SQSTM1 (Santa Cruz, Shanghai, China), anti-ATG5 (Cell Signaling Technology, Shanghai, China), anti-DJ-1 (Bioworld, Shanghai, China), anti-HSP60 (Cell Signaling Technology), anti-mitochondria (MTC02) (Abcam, Shanghai, China), anti-TGEV nucleocapsid protein (N) (Preservation of our laboratory), anti-GAPDH (Multisciences, Hangzhou, China) and HRP-conjugated secondary antibodies (Multisciences).

Chemical reagents used in this study were 3-Methyladenine (3-MA), chloroquine (CQ), and Carbonyl cyanide 3-chlorophenylhydrazone (CCCP), obtained from Sigma-Aldrich. Rapamycin was obtained from Gene Operation (Michigan, USA), and cyclosporine A (CsA) and Rotenone were obtained from Beyotime Institute of Biotechnology.

### Plasmids and transfections gene transduction

ATG5 (Autophagy related 5) and DJ-1 (deglycase 1 also calls PARK7) knockdown in IPEC-J2 cells was performed using the vector-based shRNA approach. shRNA targeting sequences against ATG5 and DJ-1 were designed using online design tools (http://rnaidesigner.lifetechnologies.com/rnaiexpress/insert.do), BLOCK-iT RNAi Designer, Life Technologies); we selected three short target sequences with the best scores for each gene. Table 1 lists the oligonucleotides used. The shRNAs were cloned into the pLVX-shRNA1 vector (Takara, Dalian, China) containing EcoRI and BamHI sites. The pmCherry-EGFP-LC3B vector was purchased from Addgene (Cambridge, MA, USA). The DNA fragment of mito-signal peptide was amplified from human genome using the primers PDHA1-F and PDHA1-R, and inserted into the lentiviral vector pLVX-mCherry-N1; the resulting plasmid was named pLVX-mitomCherry. The DNA fragment of IRES-EGFP was amplified from pIRES-EGFP-N1 and the LC3B (microtubule associated protein 1 light chain 3 beta) fragment was amplified from the cDNA of IPEC-J2 cells. The primers were IRES-F, EGFP-R and LC3B-F, LC3B-R, respectively. The amplified fragments were then overlapped with each other and inserted into the lentiviral vector pLVX-mitomCherry; the resulting plasmid was named pLVX-mitomCherry-IRES-EGFPLC3B.

**Table 1:** Primer sequences used for plasmids construction

Primers names	Type	Sequences (5′ to 3′)
PDHA-F	Forward	CGCGGGCCCGGGATCCGCCACCATGAGGAAGATGCTCGCCGC
PDHA-R	Reverse	TGCTAGCCATGGTACCGTTACGGGACGCCACCAGC
IRES-F	Forward	CGCGGGCCCGGGATCCCCCCTCTCCCTCCCCCCCCCCTAA
EGFP-F	Forward	CGGACTCAGATCTCGAGGCCACCATGGAGCAGAAACTCATCTCTGAAG
EGFP-R	Reverse	CTCGGACGGCATGATATCGAATTCTTTGTAGAGCTCATCCATGC
LC3B-F	Forward	GAGCTCTACAAAGAATTCGATATCATGCCGTCCGAGAAAACCTT
LC3B-R	Reverse	GGAGAGGGGCGGATCCTTACACAGACAACTTCATTCCG
shRNA1-DJ1-F(Control)	Forward	GATCCGAGTTCGGGAAGGATCGTTACCGAAGTAACGATCCTTCCCGAACTCTTTTTTG
shRNA1-DJ1-R(Control)	Reverse	AATTCAAAAAAGAGTTCGGGAAGGATCGTTACTTCGGTAACGATCCTTCCCGAACTCG
shRNA1-DJ1-F	Forward	GATCCGGAGATGGAGACGGTTATTCCCGAAGGAATAACCGTCTCCATCTCCTTTTTTG
shRNA1-DJ1-R	Reverse	AATTCAAAAAAGGAGATGGAGACGGTTATTCCTTCGGGAATAACCGTCTCCATCTCCG
shRNA1-ATG5-F(Control)	Forward	GATCCGAGAGTCGATACTCGTAGTATCGAAATACTACGAGTATCGACTCTCTTTTTTG
shRNA1-ATG5-R(Control)	Reverse	AATTCAAAAAAGAGAGTCGATACTCGTAGTATTTCGATACTACGAGTATCGACTCTCG
shRNA1-ATG5-F	Forward	GATCCGGAGGCAGAACCGTATTATTTCGAAAAATAATACGGTTCTGCCTCCTTTTTTG
shRNA1-ATG5-R	Reverse	AATTCAAAAAAGGAGGCAGAACCGTATTATTTTTCGAAATAATACGGTTCTGCCTCCG
N-F	Forward	CGCGGGCCCGGGATCCGCCACCATGGCCAACCAGGGACAACGT
N-R	Reverse	TCATCATCATCTTTATAATCGTTCGTTACCTCATCAATTA
Flag-R	Reverse	AGAATTATCTAGATTATTTATCATCATCATCTTTATAATC
mRFP-F	Forward	CCGCGGGCCCGGGATCCGCCACCATGATGGTGTCTAAGGGCGAA
mRFP-R	Forward	GATGAGTTTCTGCTCGGATCCATTAAGTTTGTGCCCCAG
Bcl-xL-F	Forward	GACGAGCTGTACAAGGAGAGCCGAAAGGGCCAG
Bcl-xL-R	Reverse	CGGTAGAATTATCTAGATTATTTCCGACTGAAGAGTGAGC

To construct pLVX-EGFPLC3, the EGFP-LC3 fragment was amplified from pLVX-mitomCherry-IRES-EGFPLC3B by primers EGFP-F and LC3B-R, and inserted into the vector pLVX-puro, which was linearized by BamHI and XbaI digestion. The construction of PLVX-mRFP-EGFP-LC3 was based on pLVX-EGFPLC3. Fragment mRFP was amplified from pTagRFP N1 using primers mRFP-F and mRFP-R, and then inserted into pLVX-EGFPLC3 linearized by XhoI and BamHI digestion. The construction of pLVX-mRFP-EGFP-Bcl-xL was based on PLVX-mRFP-EGFP-LC3, the fragment LC3 in pLVX-mRFP-EGFP-LC3 was digested by HpaI and XbaI, and replaced with the transmembrane structure of fragment Bcl-xL at the same site, which was amplified from human genome by primers Bcl-xL-F and Bcl-xL-R. To construct pLVX-N-Flag, the DNA fragment of TGEV N gene was amplified from TGEV-infected IPEC-J2 cells cDNA by primers N-F and N-R, subsequently, fused with Flag tag at 3′ end by primers N-F and Flag-R. Then the N-Flag fragment was inserted into the linearized pLVX-puro mentioned above.

Lentiviral production was achieved through calcium phosphate transfection of four plasmids, following the lentivirus packaging protocol [87]. For infection 1 × 10^5^ IPEC-J2 cells were plated in 9 cm^2^ dishes a day prior to infection. The day of infection the viral supernatant was supplemented with Polybrene (8 mg/mL) and added to the cells for 8 h. at a MOI (multiplicity of infection) of 1 to obtain single copy integration of the synthetic gene circuit. The cells were then expanded in DMEM with 5 μg/mL puromycin. Cell lines infected with the ATG5 and DJ-1 shRNA vectors, and the negative control (scrambled) vector-infected cells, were designated shRNA-ATG5, shRNA-DJ1 and shRNA-control, respectively.

### Autophagic and mitophagic flux detection

The principle of autophagy flux detection by mRFP-EGFP-LC3 is based on the differential stability of green and red fluorescent proteins at varying pH. The acidic environment (pH below 5) inside the lysosome quenches the fluorescent signal of EGFP, yet has much less effect on mRFP. In green/red merged images, yellow puncta (mRFP+ EGFP+) indicate autophagosomes, while red puncta (mRFP+ EGFP-) indicate autolysosomes. Rapamycin can efficiently induce autophagic flux resulting in red puncta accumulation [88, 89], while CQ can efficiently inhibit the fusion of autophagosomes and lysosomes resulting in yellow puncta accumulation [90, 91]. To detect autophagic flux in IPEC-J2 cells, we transfected pLVX-mRFP-EGFP-LC3 into IPEC-J2 cells and then infected with TGEV. To induce complete autophagy flux, the cells were treated with 100 nmol/L rapamycin for 12 hours, to inhibit the autophagy flux, the cells were treated with 50 μmol/L CQ and 100 nmol/L rapamycin for 12 hours.

The principle of mitophagy flux detection by mRFP-EGFP-Bcl-xL is similar with mRFP-EGFP-LC3. To induce complete mitophagy flux, the cells were treated with 10 nmol/L CCCP for 3 hours. On the contrast, to inhibit the mitophagy flux, the cells were treated with 50 μmol/L CQ and 10 nmol/L CCCP for 3 hours.

### Autophagy inhibition and induction, mitochondrial membrane potential stabilization, and apoptosis induction

To inhibit autophagy, cells were pre-treated with 5 mmol/L 3-MA in culture media for 12 hours prior to TGEV infection, then treated with 5 mmol/L 3-MA in fresh culture media after infection. To induce macroautophagy, cells were pre-treated with 100 nmol/L rapamycin in culture media for 12 hours prior to infection, then treated with 100 nmol/L rapamycin in fresh culture media after TGEV infection. To induce mitophagy, cells were pre-treated with 10 μmol/L CCCP in culture media for 3 hours prior to infection then 10 μmol/L CCCP in fresh culture media post TGEV infection. To inhibit mitochondrial membrane potential decrease, the cells were pre-treated with 10 μmol/L CsA, an inhibitor for mPTP (mitochondrial permeability transition pore), in culture media for 12 hours prior to TGEV infection and 10 μmol/L CsA in fresh culture media after TGEV infection. To induce apoptosis, the cells were pre-treated with 100 μmol/L Rotenone in culture media, inducing apoptosis through the mitochondrial pathway [92], for 24 hours prior to infection and 100 μmol/L Rotenone in fresh culture media after TGEV infection. Culture media in all cases was DMEM supplemented with 2% FBS.

### TGEV propagations and infections

TGEV (SHXB strain) was provided by Jiangsu Provincial Academy of Environmental Science (JAAS), and propagated in ST cells. 50% Confluent IPEC-J2 cells were inoculated with TGEV at a MOI of 5 for 1 h at 37°C. The inoculum and unattached virus were removed and fresh growth medium was added. Infected cells were analyzed after the required incubation period.

### Plaque assay

Confluent monolayers of ST cells grown in 6-well tissue culture plates were infected with 250 μL of serial tenfold dilutions of the virus suspension. After incubation for 1 h at 37°C, cells were overlaid with 0.7% Sea-Plague agarose in DMEM containing 2% FBS and incubated at 37°C. At 2 days post-infection, plaques were visualized by staining with Crystal Violet in 0.8% agar.

### Western blot analysis

Cells were lysed in RIPA buffer (50 mMTris-HCl (pH 7.4), 150 mM NaCl, 1% NP-40) containing a protease inhibitor cocktail (Thermo Scientific). Protein concentration was determined by BCA protein quantification kit (Thermo Scientific). Equal amounts of protein were separated by SDS-PAGE and electrophoretically transferred onto a PVDF membrane (Millipore, Shanghai, China). After blocking with 5% nonfat milk in Tris-buffered saline containing 0.1% Tween-20, the membrane was incubated with specific primary antibodies (1:1000), followed by incubation with appropriate horseradish peroxidase-conjugated secondary antibodies. Signals were detected using SuperSignal WestPico kit (Thermo Scientific), and subjected to Image Reader LAS-4000 imaging system (FUJIFLIM, Japan).

### Quantitative RT-PCR

For quantitative reverse transcription-polymerase chain reaction (qRT-PCR), total cellular RNA was extracted with TRIZOL (Life Technologies) and RNA was reverse-transcribed (TaKaRa, Dalian, China). qPCR was performed using the Real-Time PCR system (ABI 7500, Life Technologies, USA). Gene expression was calculated with the comparative Ct method and normalized to the endogenous levels of GAPDH. Primer sequences used for qRT-PCR are listed in Table 2.

**Table 2:** Primer sequences used for qRT-PCR

Primers names	Type	Sequences(5′ to 3′)
SCD-F	Forward	AGCTTTAAGGTGGGTGGCTC
SCD-R	Reverse	TGCTTTCGAAGCTTTGTGCC
eNOS-F	Forward	GCCTGAACAGCACAGGAGTT
eNOS-R	Reverse	ACGAGCAAAGGCACAGAAGT
DJ1-F	Forward	AGAGGAAGGGCCTCATAGCA
DJ1-R	Reverse	TCAGACCGTCCTTTTCCACG
GAPDH-F	Forward	TCATCATCTCTGCCCCTTCT
GAPDH-R	Reverse	GTCATGAGTCCCTCCACGAT

### Fluorescence microscopy

IPEC-J2 cells were transiently transfected with pmCherry-EGFP-LC3 24 h prior to virus infection. IPEC-J2 cells were transfected with pLVX-mitomCherry-IRES-EGFPLC3B or pLVX-mito-mCherry and cultured 1 week in DMEM/10% FBS containing 3 μg/mL puromycin. Cells were grown on coverslips and fixed with 4% paraformaldehyde for observation with a Zeiss LSM710 confocal microscope (Carl Zeiss, Germany), the images were analyzed using ZEN 2012 (Blue edition) (Carl Zeiss).

### Flow cytometry

Apoptotic cell death was detected by Annexin V/propidium iodide (PI) staining assay (Miltenyi Biotec, Shanghai, China) according to the manufacturer's protocols. Briefly, cells were harvested and washed once with PBS, then resuspended in 100 μL binding buffer followed by incubation with 10 μL Annexin V per test for 20 min. Cells was washed again, suspended in 500 μL of binding buffer and 5 μL PI per test was added and immediately analysis by flow cytometry (FACS) (Becton, Dickinson and Company, USA).

Dysfunctional mitochondria were monitored by fluorescence levels upon staining with 100 nM MitoTracker Green FM (total mitochondria) (Life Technologies) and 500 nM MitoTracker Red CMXRos (functional mitochondria) (Life Technologies) for 25 min at 37°C, after which, cells washed with PBS and analyzed at FL-1 and FL-3 by FACS. Mitochondrial mass was measured by fluorescence levels upon staining with 100 nM Mitotracker Green FM for 25 min at 37°C, after which, cells were then washed with PBS and analyzed at FL-1 by FACS. In parallel, the mean of mCherry in mito-mCherry positive cells were also used to detect the mass of mitochondria at FL-3 by FACS.

To detect the degree of mitochondria injury after TGEV infection, the mitochondrial membrane potential (Δψ) was monitored by fluorescence levels upon staining with 1μg/mL Rhodamine 123 for 25 min at 37°C. After staining, cells were then washed with PBS and analyzed at FL-1 by FACS.

To detect the degree of autophagy after TGEV infection, cells were incubated in Cyto-ID (1 μL Cyto-ID/1mL cell culture medium without phenol red indicator) for 30 minutes and washed prior to analysis by FACS. The Cyto-ID assay (Enzo Life Sciences, Shanghai, China) incorporates a 488 nm-excitable green fluorescent detection reagent that specifically fluoresces in autophagic vesicles. An increase in the number of autophagic vesicles labeled with green is detected as an increase in fluorescence in the FL-1 channel.

The intracellular production of ROS and mitochondrial ROS was measured by using the fluorescent probe 6-carboxy-2′, 7′-dichorodihydrofluorescein diacetate (DCFH-DA) and Dihydroethidium (DHE), respectively. After treatment or TGEV infection, cells were incubated with 10 μM DCFH-DA or 40 μM DHE in serum-free medium for 10 minutes at 37°C. Afterwards, cells were harvested and resuspended in 500 μL of PBS, and DCF and DHE fluorescence was measured by FACS. All data were analyzed using FlowJo software (Version 7.6.5, Tree Star Inc., Ashland, Oregon).

### Cell fractionation

Mitochondrial and cytoplasmic proteins were separated using a Mitochondria/Cytosol Fractionation Kit (Beyotime Institute of Biotechnology) according to the manufacturer's protocol. Briefly, 5 × 10^7^ cells were harvested and washed twice with ice-cold PBS, cells were then incubated in 500 μL ice-cold mitochondrial lysis buffer on ice for 10 min. Using a glass Dounce homogenizer with a tight pestle, the cell suspension was then homogenized for 50 strokes on ice. The homogenate was centrifuged at 800 g for 10 min at 4°C to remove any unbroken cells. The supernatant was further centrifuged at 12000 g for 10 min at 4°C to remove the mitochondrial fraction (pellet) and cytoplasmic proteins (supernatant). Proteins were quantified and subjected to immune blotting.

### Statistics

Data are presented as means ± SEM. Statistical analysis was performed using Statistical Program for Social Sciences (SPSS) 16.0. Significance was determined by Analysis of Variance (ANOVA). A *P* value less than 0.05 was considered to be significant, and less than 0.01 was considered to be highly significant.

## SUPPLEMENTARY MATERIALS FIGURES


