# Supplementary material for: Mitophagy in TGEV infection counteracts oxidative stress and apoptosis
Source: Oncotarget. 2016 Mar 24;7(19):27122–41. doi: 10.18632/oncotarget.8345 (PMC5053637; doi:10.18632/oncotarget.8345)
Supplement: Supplementary file 1 [file oncotarget-07-27122-s001.pdf]

Mitophagy in TGEV infection counteracts oxidative stress and apoptosis

Supplementary Material

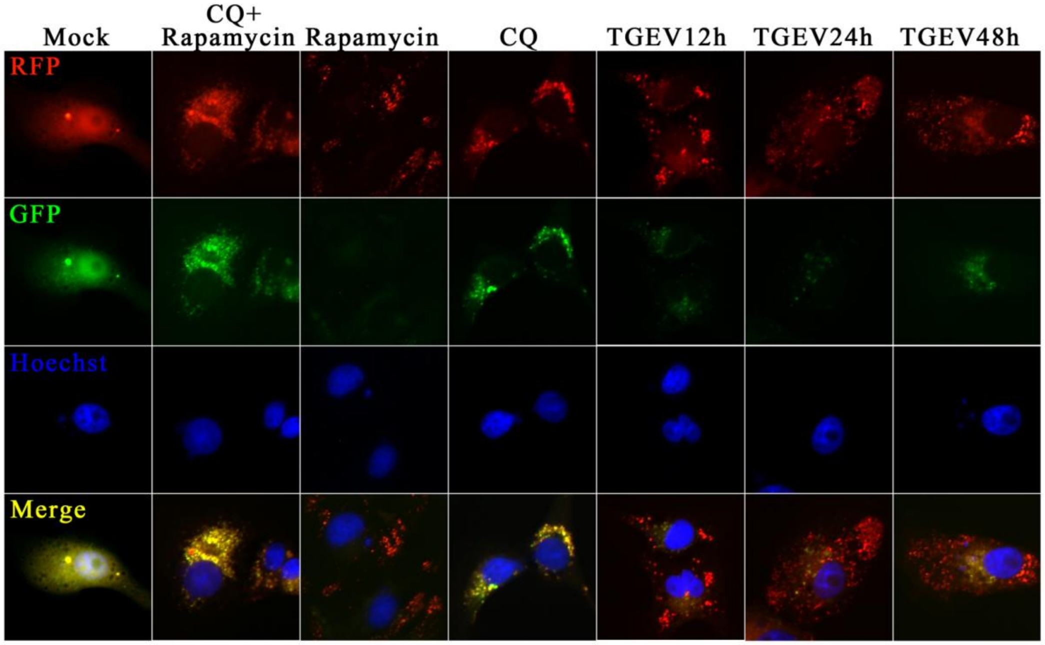

Figure S1: TGEV induce complete autophagy. The IPEC-J2 cells stably expressing mRFP-EGFP-LC3 were treated with rapamycin, CQ, or rapamycin + CQ for 12h, or infected with TGEV for 12 h, 24 h, 48 h. these cells were stained with Hoechst 33342 (blue color in the images) and observed by fluorescence microscopy in living cells. The yellow puncta indicate incomplete autophagic flux, the red puncta indicate complete autophagic flux.

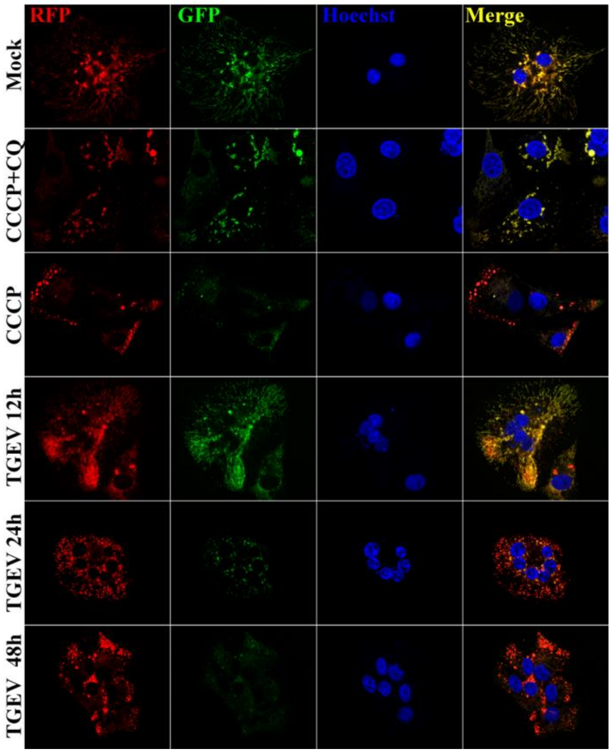

Figure S2: TGEV induce complete mitophagy. The IPEC-J2 cells stably expressing mRFP-EGFP-Bcl-xL were treated with CCCP, or CCCP + CQ for 12h, or infected with TGEV for 12 h, 24 h, 48 h. These cells were stained with Hoechst 33342 (blue color in the images) and observed by confocal fluorescence microscopy in living cells. The yellow puncta indicate normal mitochondria or incomplete mitophagy; the red puncta indicate complete mitophagy.

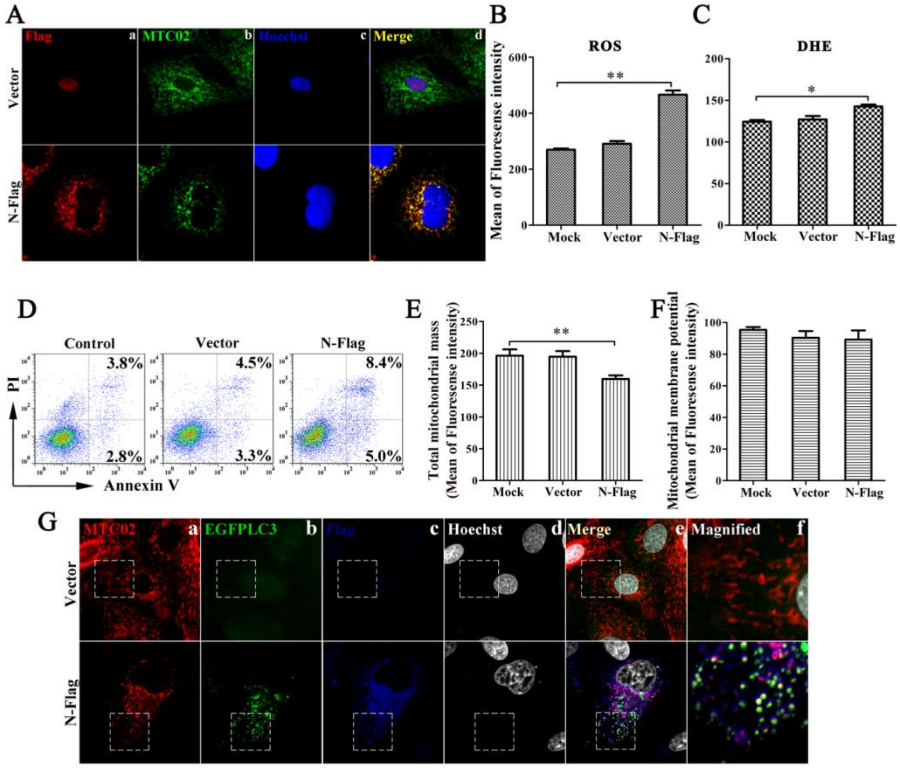

Figure S3: TGEV Nucleocapsid protein induce moderate mitochondrial injury and mitophagy. A: The stably expressing N-Flag or vector cells were immunostained with antibodies specific to Flag (red) and MTC02 (green), respectively. Nuclei were stained with Hoechst 33342 (blue). The images were observed by confocal fluorescence microscopy. **B to F:** The stably expressing N-Flag, vector and mock cells with similar passage times were digested and divided to 5 equal parts, which were stained by DCFH-DA (**B**), DHE (**C**), Annexin V-PI (**D**), MitoTracker Green (**E**) and Rhodamine 123 (**F**), respectively. After stain, the cells were tested and analyzed by flow cytometry. The data represent the mean ± SD of three independent experiments. One-way ANOVA; *, $P < 0.05$; **, $P < 0.01$. **G:** The stably expressing N-Flag or vector cells were transfected with pLVX-EGFP-LC3 lentiviral and were screened by blasticidin one week. Cells were immunostained with antibodies specific to Flag (blue) and MTC02 (red), respectively. Nuclei were stained with Hoechst 33342 (white). The images were observed by confocal fluorescence microscopy. In the images, the colors correspond to nuclei (white), Flag (blue), EGFP-LC3 (green), and MTC02 (red). Co-localized signals for Flag, EGFP-LC3 and MTC02 are white in the cytoplasm of the merged images. Higher magnification images represent the regions enclosed in white dotted squares.
